# Practical Anatomy

**Published:** 1883-08-20

**Authors:** 


### EDITORIALS AND MISCELLANEOUS.

### *PRACTICAL ANATOMY.*

The bill before the Georgia Legislature to facilitate the study of Practical Anatomy has again failed. That a great State should demand of the Surgeon a knowledge of Anatomy, prosecuting him for mal-practice if he fails to understand it, and yet make penal all efforts to obtain the required knowledge, is an inconsistency unworthy of our boasted civilization.

